# Germline Missense Variants in *BRCA1*: New Trends and Challenges for Clinical Annotation

**DOI:** 10.3390/cancers11040522

**Published:** 2019-04-12

**Authors:** Volha A. Golubeva, Thales C. Nepomuceno, Alvaro N. A. Monteiro

**Affiliations:** Cancer Epidemiology Program, H. Lee Moffitt Cancer Center and Research Institute, Tampa, FL 33612, USA; golubeva.3@osu.edu (V.A.G.); thales.cn@gmail.com (T.C.N.)

**Keywords:** *BRCA1*, variants of uncertain clinical significance, VUS, germline variants, hereditary breast and ovarian cancer, breast cancer, genetic testing, ovarian cancer, variant classification, clinical annotation

## Abstract

Genetic testing allows for the identification of germline DNA variations, which are associated with a significant increase in the risk of developing breast cancer (BC) and ovarian cancer (OC). Detection of a *BRCA1* or *BRCA2* pathogenic variant triggers several clinical management actions, which may include increased surveillance and prophylactic surgery for healthy carriers or treatment with the PARP inhibitor therapy for carriers diagnosed with cancer. Thus, standardized validated criteria for the annotation of *BRCA1* and *BRCA2* variants according to their pathogenicity are necessary to support clinical decision-making and ensure improved outcomes. Upon detection, variants whose pathogenicity can be inferred by the genetic code are typically classified as pathogenic, likely pathogenic, likely benign, or benign. Variants whose impact on function cannot be directly inferred by the genetic code are labeled as variants of uncertain clinical significance (VUS) and are evaluated by multifactorial likelihood models that use personal and family history of cancer, segregation data, prediction tools, and co-occurrence with a pathogenic *BRCA* variant. Missense variants, coding alterations that replace a single amino acid residue with another, are a class of variants for which determination of clinical relevance is particularly challenging. Here, we discuss current issues in the missense variant classification by following a typical life cycle of a *BRCA1* missense variant through detection, annotation and information dissemination. Advances in massively parallel sequencing have led to a substantial increase in VUS findings. Although the comprehensive assessment and classification of missense variants according to their pathogenicity remains the bottleneck, new developments in functional analysis, high throughput assays, data sharing, and statistical models are rapidly changing this scenario.

## 1. Introduction

Breast cancer (BC) is the leading cause of cancer death among women worldwide [1]. Common risk factors for breast cancer include breast density, older age, family or personal history of breast cancer, lifetime hormonal exposure of breast tissue (early menarche, late menopause, and hormone use) and behavioral factors among others (smoking, alcohol consumption, obesity, physical inactivity, poor diet, stress and anxiety) [2]. According to the International Agency for Research on Cancer, ovarian cancer (OC) ranks eighth among cancer deaths in women worldwide [1]. Risk factors for OC are linked to older age, obesity and personal or family history of OC. Moreover, hormone therapy, late full-term pregnancy (after 35 years), or not having given birth also increases the risk of OC development [3].

The Hereditary Breast and Ovarian Cancer (HBOC) syndrome [OMIM 604370 and 612555] is a *BRCA1*- or *BRCA2*-linked genetic disorder associated with a high risk of developing BC and OC. Hereditary breast cancers constitute about 5–10% of all BC cases but pathogenic variants in the high-risk (RR ≥ 4) breast cancer susceptibility genes *BRCA1* [OMIM 113705] and *BRCA2* [OMIM 600185] account for approximately 50% (0.7–29% *BRCA1* and 1.5–25% *BRCA2*) of all hereditary breast cancers [4]. Germline pathogenic variants in *BRCA1* and *BRCA2* account for about 14% of all epithelial ovarian cancers and 16.6% of high-grade serous ovarian cancers [5]. Carriers of a pathogenic variant in the *BRCA1* gene have a 72% cumulative risk up to 80 years of developing BC (5-fold increase), and 44% for OC (30-fold increase) [6].

The U.S. National Comprehensive Cancer Network (NCCN) guidelines advise who should be tested for *BRCA1/2* pathogenic variants in the U.S. Among the predictors for genetic counseling referral are the diagnosis of BC before the age of 50 (60 for triple-negative BC), one sole case of OC, male BC, known family history of BC, OC and/or pancreatic cancer on the same side of the family and others [7]. Although guidelines vary across different countries, they follow a similar trend with genetic counseling as a pre-requisite for testing and family history of cancer being central to the selection of which individuals should be tested [8]. For a comparison of guidelines in Europe (UK, France, Netherlands, and Germany) and in Latin America the reader is referred to in-depth papers on the subject [8,9,10].

**Figure 1:**
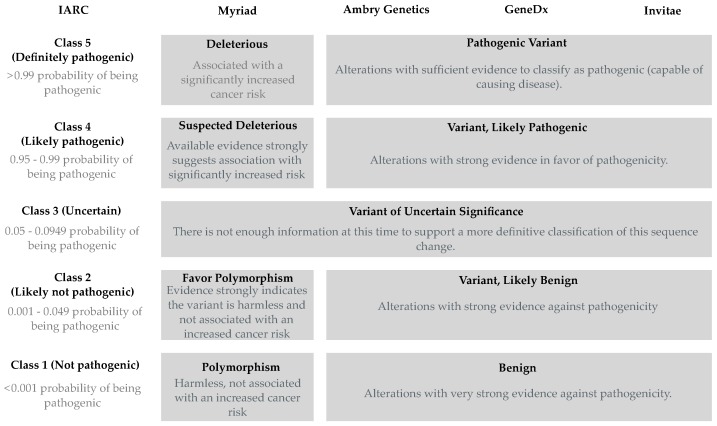
Variations of the five-tier system used to convey information about a variant in *BRCA1*. Classification recommended by the IARC Unclassified Genetic Variants Working Group (IARC) and endorsed by the Evidence-based Network for the Interpretation of Germline Mutant Alleles (ENIGMA) Consortium, and reported by select commercial testing companies in the U.S.

In the U.S., outcomes from commercial sequencing-based genetic testing typically range from findings of a known pathogenic variant, a variant of uncertain clinical significance (VUS), no changes (from reference sequence), or a known non-pathogenic (benign) variant organized in a five-tier system (see Figure 1 for a sample of commercial testing results) [11]. Detection of a pathogenic variant in the proband’s *BRCA1* gene will prompt specific clinical responses. Clinicians may act by adjusting health recommendations to their at-risk patients and family members, as well as altering their patients’ cancer surveillance and treatment. In addition, the identification of an individual carrying a non-pathogenic variant or reference sequence in a family linked to a pathogenic variant in *BRCA1* is also a benefit of genetic testing. Because acting on clinical recommendations tailored for carriers of pathogenic variants may substantially alter outcomes, robust and accurate methods of classification of variants are critical. We identify annotation/classification as a rate-limiting step for the implementation of precision medicine in breast and ovarian cancer care, and review current approaches to eliminate this bottleneck.

## 2. *BRCA1* Variant Detection: From First Variants to New Trends

### 2.1. Detection of the First Germline Variants in BRCA1

**Figure 2:**
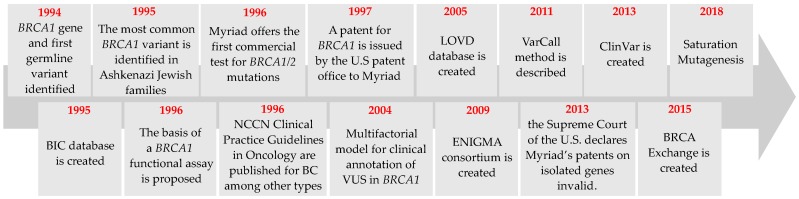
*BRCA1* timeline. BRCA1-related events in the last three decades. Depicted events are discussed throughout this review.

The first germline pathogenic variants in *BRCA1* were identified in 1994 (Figure 2), when Miki et al. analyzed sequences from patients’ complementary DNA (cDNA) clones, hybrid-selected sequences, and amplified polymerase chain reaction (PCR) products [12]. The combination of these methods allowed the construction of a composite, full-length *BRCA1* cDNA. The four variant alleles were an 11-base pair (bp) deletion in exon 2 (p.Cys24Serfs; c.69_79delGTGTCCCATCT), a nonsense variant in coding exon 11 (p.Gln1313Ter; c.3937C>T), a single nucleotide insertion in coding exon 20 (p.Gln1756Profs; c.5266dupC), and a missense variant in coding exon 21 (p.Metl775Arg; c.5324T>G). Since then, more than 10,000 unique germline variants have been detected in *BRCA1* (BRCA Exchange; https://brcaexchange.org/) [13].

Initially, the *BRCA1* locus region was shown to be lost in sporadic cases of breast and ovarian cancers, prompting the investigation of a putative role of *BRCA1* as a driver gene in sporadic cases of BC and OC [14,15,16,17]. However, inactivating somatic mutations in *BRCA1* were shown to be present in a small number of breast (1.6%) and ovarian (3.5%) tumors unselected for family history [18].

### 2.2. Commercial Testing

In the mid-90s, Myriad Genetics, Inc. was awarded a patent for *BRCA1* and *BRCA2* genetic testing by the U.S. Patent Office (Figure 2). In a few years, demand for genetic testing for HBOC increased significantly, and by 2002 hereditary cancers had surpassed all pediatric conditions combined in demand for genetic counseling time in Ontario [19]. Myriad held the U.S. patent for approximately 13 years until the American Civil Liberties Union (ACLU), the Association for Molecular Pathology (AMP) and the American College of Medical Genetics (ACMG) challenged its legality and constitutionality. After four years of litigation (from May 2009 to June 2013), the Supreme Court of the United States decided that isolated genomic DNA is not patent-eligible as it was considered a product of nature, however the cDNA, which is synthetized in the laboratory is patent-eligible [20,21]. The decision paved the way for other companies to offer genetic testing for *BRCA1/2*.

With the advances in variant detection technologies, the use of Direct-to-Consumer (DTC) genetic testing has increased, creating a new niche in personalized medicine. Although testing for *BRCA1* and *BRCA2* founder pathogenic variants has been available for many years [22,23,24,25], the U.S. Food and Drug Administration recently authorized the use of DTC testing for the three most common Ashkenazi Jewish pathogenic variants in *BRCA1* and *BRCA2* genes [26]. Individuals interested in this three-site panel test can purchase a saliva-based kit from a commercial company, such as 23andme, which makes the result available directly to the consumer. The availability of the DTC genetic testing has potential benefits as it may increase awareness and allow easier access to testing which can lead to early identification of individuals at risk. However, the problems may arise during the interpretation of the results with significant implications for intervention (i.e., lifestyle change, prophylactic surgery, and chemoprevention) in consumers who receive the DTC genetic information, in the absence of adequate genetic counseling. There is a risk of anxiety and distress for individuals receiving test results without understanding its implications. They may also make incorrect clinical decisions based on results they receive from DTC vendors. Importantly, DTC testing may also provide a false guarantee to consumers who receive negative results. Some consumers may be unaware that the absence of those three pathogenic variants does not eliminate the possibility that the risk of BC is accounted for by other alleles (pathogenic variants in other positions, genes, or non-coding regions).

Another trend encompassing not only personalized medicine, but also public health, is aimed at improving the HBOC detection rates by screening all women regardless of family history status. This new trend, also known as population-based *BRCA* testing, has been proposed [27]. Among the general cancer-free population, family history of HBOC has remained the major factor for referral to genetic testing, even though approximately half of the carriers of *BRCA* pathogenic variants does not have a significant family history [27].

### 2.3. Genetic Testing Developments

Sanger sequencing of a series of amplicons covering exons and intron-exon borders was initially the preferred method of academic and commercial testing for *BRCA1* and *BRCA2* as it provided fast and accurate results [28,29,30]. Adoption of massively parallel sequencing allowed for a cost-efficient screening of multiple gene panels, exomes, and genomes. More comprehensive testing, involving a large set of genes, is clinically beneficial to patients who are suspected of having the HBOC syndrome, but may not be linked to *BRCA1* or *BRCA2*. However, the likelihood of receiving a VUS result is considerably higher because the clinical impact of many rare variants typically cannot be determined and because for several genes their association with disease has not yet been firmly established. For a detailed review on multigene panel testing availability, clinical and analytical validity and clinical utility the reader is referred to [31,32].

However, amplicon-based Sanger sequencing or short-read lengths from massively parallel sequencing do not reliably detect large genomic rearrangements (LGR). LGR have been identified in multiple HBOC families and are estimated to be responsible for 5–10% of all *BRCA1/2* pathogenic variants [33]. *BRCA1* gene is an *Alu*-enriched genomic region, representing more than 40% of its intronic sequence and this recombinogenic feature has been proposed to underlie the relatively high *BRCA1* LGR frequency in HBOC families [34]. However, different studies point to large differences in prevalence of *BRCA1* rearrangements across different populations. LRG in *BRCA1* and *BRCA2* ranges from very rare among Canadian, French, and Finish families [35,36,37] to common among families in northern Italy and Portugal [38,39]. The multiplex ligation-dependent probe amplification (MLPA) is the most commonly used method for the identification of these alterations [40].

### 2.4. The Spectrum of BRCA1 Variants across the World

Knowledge of the prevalence of *BRCA1* pathogenic variants across different populations is important for planning cost-effective preventive interventions and for the design of low-cost testing panels in developing countries. The estimated prevalence of *BRCA1* pathogenic variants in an unselected population is better characterized in non-Hispanic whites from the United States, United Kingdom and Canada (carrier frequency ranging from 0.1 to 0.3%) than in other ethnic groups [41,42]. According to the Exome Variant Server for the NHLBI GO Exome Sequencing Project (*n* = 2203 African-Americans and 4300 European-Americans; http://evs.gs.washington.edu/EVS/), the frequency of *BRCA1* pathogenic variants in an unselected population is 0.65% and 0.51% in European and African Americans, respectively. Further, data derived from the Exome Aggregation Consortium (ExAC; *n* = 60,706; http://exac.broadinstitute.org/) indicates that frequencies for *BRCA1* pathogenic variants vary among African (0.00%), Latino (0.04%), East Asian (0.1%) and South Asian (0.2%). When it comes to individuals with Ashkenazi ancestry, the prevalence rises to 1.2% [42,43,44,45]. These studies may underestimate the prevalence of *BRCA1* pathogenic variants in the general population, as they do not include large genomic rearrangements or VUS.

Among BC patients unselected for family cancer history, the prevalence of *BRCA1* pathogenic variants varies from 0.5% to 3.1% in whites from the U.S., UK and Australia. Asian Americans have a frequency of 0.5%, while Asian populations (including Chinese, Japanese, Philippines, Pakistanis and Korean individuals) present a prevalence ranging from 0.8% to 3.9%. Additionally, African Americans and Hispanic Americans present a frequency of 1.4% and 3.5%, respectively [46].

The prevalence of *BRCA1* pathogenic variants is much higher in particular groups that share a common ancestor, which carried a founder mutation. The best characterized *BRCA1* founder pathogenic variants are c.5266dupC (p.Gln1756Profs, a.k.a. 5282insC) and c.68_69delAG (p.Glu23Valfs, a.k.a. 185delAG) found in Ashkenazi Jewish individuals in the U.S., but other distinct founder pathogenic variants have been found in French Canadians (c.4327C>T, p.Arg1443Ter; a.k.a. 4446C>T), French from North-Eastern France (c.3481_3491delGAAGATACTAG, p.Glu1161Phefs, a.k.a. 3600del11; and c.5266dupC) [47,48], Central Eastern Europeans (c.181T>G, p.Cys61Gly; a.k.a. 300T>G; c.4043delG, p.Gly1348Glufs, a.k.a. 4162delG), Mexicans (ex9-12del), Brazilians (c.5266dupC; a.k.a. 5382insC) and Colombians (c.3331_3334delCAAG, p.Gln1111Asnfs, a.k.a. c.3450_3453delCAAG; and c.5123C>A, p.Ala1708Glu) [49,50,51,52,53,54].

The identification of founder mutations in ethnic groups or specific populations can facilitate ancestry-informed genetic test and enable cost-effective genetic screenings. It has been proposed that the Ashkenazi Jewish population in the U.S. and UK would be a starting point for the implementation of a population-based screening for *BRCA1* and *BRCA2* [27,55,56,57]. In that specific population, both *BRCA1* (c.5266dupC and c.68_69delAG) and *BRCA2* (c.5946delT; a.k.a. 6174delT) founder mutations account for 90% of all pathogenic variants [56]. A cost-effective approach was developed to help identify recurrent pathogenic variants in Hispanic/Latino populations [53].

## 3. *BRCA1* Variant Annotation and Classification

### 3.1. Definitions and Limitations

For the purposes of this review we make a distinction between annotation, the process of adding evidence from multiple sources to a variant, and classification. Importantly, a variant may be heavily annotated and yet remain unclassified according to its pathogenicity. Here, we define classification as the process of assigning a variant to one of the IARC Classes either through a rule-base (e.g., variants whose impact on function can be inferred from the genetic code) or a multifactorial statistical model [11,58,59,60]. Annotated variants that are assessed by the multifactorial model but do not reach statistical thresholds for classification as benign (Class 1), likely benign (Class 2), likely pathogenic (Class 4) or pathogenic (Class 5) remain as Class 3 (still uncertain) (Figure 1).

Several important points need to be considered when interpreting classification results for variants. First, although many clinical and commercial laboratories use a similar five-tier scale (Figure 1), statistical thresholds and types of evidence considered in the model may differ significantly [61,62]. Second, for the multifactorial model it is important to stress that the model only discriminates high-risk (comparable to truncating variants) variants from variants that do not confer high-risk [59]. A common misconception is that the five-tier class system reflects different levels of risk. For example, a Class 4 variant is expected to confer a similar high-risk to a Class 5 variant, and the difference in class reflects the strength of evidence and not the levels of risk. Two-stage reporting systems have been proposed with the first stage establishing pathogenicity of the variant and the second stage would denote the severity or clinical consequence (high, moderate or low risk), but have not been implemented. Third, there are no penetrance estimates for BRCA1 missense variants as a group, and the underlying assumption that missense variants, which disable protein function, will have a comparable effect may not be correct [63]. Finally, penetrance estimates using families with multiple cases selected for linkage analysis and positional cloning are likely to represent inflated estimates when compared to the general population. For a discussion of the multifactorial model the non-specialist the reader is referred to Lindor et al. [64]. For a detailed discussion of pitfalls and recommendations for managing variants of uncertain clinical significance, please see Eccles et al. [65].

### 3.2. Mutational Landscape

**Figure 3:**
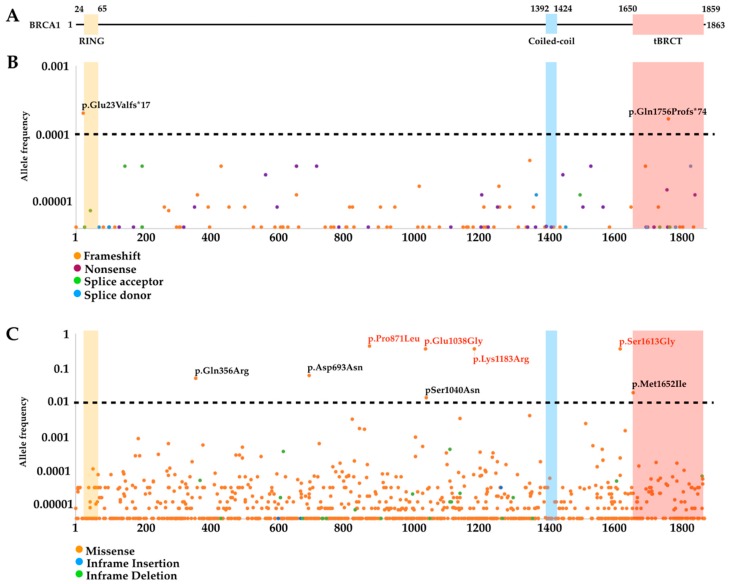
*BRCA1* allele frequency. (**A**) Diagram of BRCA1 showing the RING-finger, coiled-coil and tandem (tBRCT) domains represented by the yellow, blue and pink backgrounds respectively. (**B**,**C**) Graphic representation of the allele frequency of *BRCA1* (Ensembl transcript ENST00000357654.7) germline variants (HGVS nomenclature) deposited on the genome aggregation database (gnomAD) with data for individuals not selected for family or personal history of cancer [72] showing (**B**) frame shift (orange), nonsense (purple), splice acceptor (green) and donor (blue) variants; or (**C**) missense (orange), in frame insertion (blue) and deletion (green) variants.

Pathogenic variants, including nonsense and frame shift changes, in *BRCA1* are scattered along its sequence without a specific hotspot (Figure 3). Alterations in the coding region can generate several different outcomes on the protein product which, in some cases, can be used to infer a functional impact [66,67]. Because loss-of-function variants of *BRCA1* are associated with a significantly increased risk of cancer, frame shift and nonsense mutations (Figure 3A), whose impact on the protein can be easily inferred from the genetic code, are classified as pathogenic alterations [66]. Nonsense variants (Figure 3A) are characterized by the introduction of a premature stop codon. Frame shift occurs when the insertion or deletion of nucleotides disrupt the original reading frame. Usually, this alteration also leads to a premature stop codon. Because the nonsense variant p.Tyr1853*, which leads to the loss of the last 11 amino acid residues in BRCA1, is associated with increased risk, any premature stop codon upstream of this codon (generated by a nonsense or frame shift mutations) is likely to constitute a pathogenic variant [68,69,70,71]. In general, the same interpretation can be made toward alterations on canonical acceptor or donor splice sites, which are usually classified as pathogenic due to the predicted truncation of the full-length protein. However, caution is warranted as it has been proposed that alternative slicing transcripts with sufficient tumor suppressor activity can arise as a compensatory mechanism to overcome the absence of full-length transcripts [67].

As shown in Figure 3A, frame shift variants are more frequent than nonsense and splice site alterations. According to the Genome Aggregation Database (gnomAD; *n* = 125,748 exome and 15,708 whole-genome sequences; https://gnomad.broadinstitute.org/), the highest allele frequencies observed for p.Glu23Valfs (c.68_69delAG) (*f* = 0.0001987) and p.Gln1756Profs (c.5266dupC) (*f* = 0.0001623) variants. Nonsense alleles are rarer, with the most common p.Lys654Ter (c.1960A>T) and p.Ser713Ter (c.2138C>G) showing a frequency of 0.0000323 [72]. The functional interpretation of missense and small in frame insertions/deletions offer a more challenging scenario (Figure 3B). The exact impact of a single amino acid residue change or in frame insertion/deletion of small regions in the biochemical and biophysical properties of the protein is difficult to predict. Missense variants are the most frequent type of *BRCA1* germline alterations (Figure 3B). Some of these variants occur with an allele frequency >1% and are therefore considered non-pathogenic or benign (e.g., p.Gln356Arg, c.1067A>G; p.Asp693Asn, c.2077G>A; p.Pro871Leu, c.2612C>T; p.Glu1038Gly, c.3113A>G; p.Ser1040Asn, c.3119G>A; p.Lys1183Arg, c.3548A>G; p.Ser1613Gly, c.4837A>G; and p.Met1652Ile, c.4956G>A) [72]. On the other hand, the vast majority of *BRCA1* missense variants have low allele frequencies (<1%). Although representing a very small fraction of all missense variants, some single nucleotide substitutions that result in amino acid changes (missense variant) may also affect splicing (e.g., *BRCA1* p.R1495M, c.4484G>T; *BRCA2* p.I2675V, c.8023A>G), and their effect on function may be due to the missense change or the splicing, or both. In frame insertions/deletions are the least frequent class of variants (<0.001) [72]. The use of whole-genome sequencing for screening of individuals suspected of being carriers, which has led to the findings of VUS in non-coding regions, has raised a new challenge for annotation and classification. For intronic and untranslated regions, gnomAD now includes ~1000 VUS.

### 3.3. Variants of Uncertain Significance (VUS)

Variants whose impact on function cannot be directly inferred by the genetic code are categorized as VUS and are evaluated by multifactorial likelihood models that use personal and family history of cancer, segregation data, prediction tools, and co-occurrence with a pathogenic BRCA1 variant [58,59,64]. VUS are primarily missense and splicing variants. However, the low frequency of the majority of germline missense variants in the population (<1/10,000; Figure 3B) impairs clinical annotation derived from linkage analysis. The limited access to family history and genetic information impedes the use of likelihood models. The current challenge of the clinical annotation of VUS lies on the inability to assess the pathogenicity of a variant without family or epidemiological data. In this context, the role of functional data to support the VUS classification has become critical.

### 3.4. Functional Assays

Functional assays represent an important tool for assessing the impact of a single amino acid residue change on a specific function or the integrity of a specific protein functional domain. *BRCA1* encodes a homonymous protein composed by 1863 amino acid residues that comprises a RING-finger domain at the amino-terminus, a coiled-coil motif at the central region and two BRCT domains in tandem (tBRCT) on its carboxy-terminus (Figure 3). BRCA1 acts as a scaffold for the recruitment of several other proteins associated with genomic integrity maintenance, such as cell cycle control, DNA damage repair and transcriptional regulation [73].

Throughout the years, several functional assays have been proposed to evaluate the impact of variants on BRCA1 biological roles and biochemical properties (E3 ubiquitin ligase activity, cell cycle control, genotoxic agent sensibility, phosphopeptide binding, protease sensitivity and others) [74,75]. For variants in the carboxy-terminus of BRCA1 all assays display high specificity (80–100%) but more variable sensitivity (63–100%) with lower sensitivity seen in yeast–based presumably due to pathogenic variants which are stable at lower temperatures [76]. The high sensitivity and specificity found for most mammalian-based assays allow for the use of functional data in classifying VUS.

The first proposed functional assay for BRCA1 was based on the observation that the tBRCT domain integrity correlates to the transactivation activity (TA) of its C-terminal region [70]. The assay was further validated by the functional impact of known cancer-associated variants and BRCA1 TA. Over the last 15 years, more than 300 variants enclosed within the 1396–1863 amino acid residues (all known missense variants in the C-terminal region of the BRCA1) were evaluated [76,77]. By comparing the results of BRCA1 TA with a reference panel of 49 missense variants classified by multifactorial models, using a Bayesian hierarchical model (VarCall) to predict the likelihood of pathogenicity, we observed a 1.0 sensitivity and 1.0 specificity (lower bound of 95% confidence interval = 0.78 and 0.84, respectively), demonstrating that this approach can classify VUS with confidence [76].

Recently, two saturation mutagenesis-based high-throughput approaches evaluated the impact of 1056 and 3893 *BRCA1* variants in exons 2–7 and 15–24, respectively [78,79]. Each study used a distinct biological role of BRCA1. Starita et al. [79] interrogated the impact of alteration on the amino-terminus of BRCA1 on its role on Homology-directed Repair (HDR) while Findlay et al. [78] based their assay on the requirement of a functional *BRCA1* for viability in a near-haploid human cell line (HAP1). These studies demonstrated the feasibility of this approach and reported an excellent agreement with the available clinical data in the ClinVar database [78]. The high sensitivity and specificity found for most mammalian-based assays allow for the use of functional data in classifying VUS.

## 4. BRCA1 Variant Databases and Information Dissemination

An important factor in implementing precision medicine approaches for BC and OC is the availability of freely accessible and accurate information on variants. Databases that contain information on *BRCA1* variants come in several forms. For example, several databases that aggregate information from exome or genome sequencing in populations unselected for personal or family history can be used to estimate the frequency of variants in diverse populations. They include the Exome Variant Viewer from the Exome Sequencing Project (http://evs.gs.washington.edu/EVS/), the Exome Aggregation Consortium (ExAC; http://exac.broadinstitute.org/) and the Genome Aggregation database (https://gnomad.broadinstitute.org/). However, these databases do not contain variant classification, although some may provide in silico prediction tools to predict the potential impact of the variant on the function of the protein.

Some databases aggregate data from clinical testing, in which most individuals tested have a personal or family history of disease or are suspected of being carriers of a predisposition gene. ClinVar (https://www.ncbi.nlm.nih.gov/clinvar/) is the most widely used clinical database and aggregates information about genomic variation in any gene and its relationship to human disease. It displays variant classifications suggested by the depositor as well as those curated by expert panels.

There are several databases that are specific for *BRCA1* and *BRCA2*. The Breast Cancer Information Core database (BIC; https://research.nhgri.nih.gov/bic/) was the first centralized database for protocols, primers, and variants found in *BRCA1/2* during genetic testing [80,81,82]. Importantly, the frequency of variants in this database should not be taken as an indication of allele frequency in the general population because most individuals tested were selected for family or personal history. The BIC database contains information about pathogenicity, as contributed by the data depositor and lately, as curated by the ENIGMA consortium. The BRCA1 Circos interactive tool (https://research.nhgri.nih.gov/bic/circos/) is available through the BIC (NHGRI) webpage and compiles functional data for missense variants in BRCA1 [74]. Information about VUS that have recently been classified by the multifactorial statistical model [58,59] can be found in the Ex-VUS LOVD database (http://hci-exlovd.hci.utah.edu/home.php), and a compilation of scientific literature referring to *BRCA1/2* variants is available in the Global Variome shared database (https://databases.lovd.nl/shared/variants/BRCA1?search_VariantOnGenome/Genetic_origin=vitro), also in the LOVD format.

The BIC database is currently phasing out as all its variant information has been transferred to the BRCA Exchange (https://brcaexchange.org/) [13]. The BRCA Exchange, part of the BRCA Challenge, is a data-sharing initiative by the Global Alliance for Genomics and Health (GA4GH) and aggregates *BRCA1* and *BRCA2* data on the impact of genetic variation on cancer risk. It contains over 20,000 variants with over 6000 with expert classifications based on shared information from existing clinical and population databases discussed above (BIC, ClinVar, LOVD, gnomAD, etc.) provided by the ENIGMA consortium [83]. Importantly, the BRCA Exchange database is now available through a phone app. The BRCA Exchange is a significant step towards a wide dissemination of accurate information on *BRCA1* variants.

In addition to large aggregating international databases, there are also several national efforts in building specific population databases, such as The Singapore Human Mutation And Polymorphism Database (http://shmpd.bii.a-star.edu.sg) [84], the Kathleen Cuningham Foundation Consortium for research into Familial Breast cancer (kConFab; http://www.kconfab.org) [85], the Diagnostic Mutation Database (DMuDB; https://secure.dmudb.net) and BRCA Share^TM^ (http://www.umd.be/BRCA1/formerly) UMD-BRCA1 database (http://www.umd.be/BRCA1) [86], and the database for Brazilian genomic variants ABraOM (http://abraom.ib.usp.br/) [87], but access rules may vary.

## 5. Conclusions

Since the identification of *BRCA1* in 1994, the screening of germline pathogenic variants that might increase an individual’s risk of BC and OC has become mainstream in clinical practice. The advances in DNA sequencing have enabled rapid, accurate, and cost-effective detection of variants in *BRCA1*. Rapid development has also occurred in the dissemination of information on variants with the recent deployment of the BRCA Exchange. However, the main challenge for the use of this data to improve outcomes is the classification of variants according to their clinical relevance, in particular rare variants for which population and clinical information is lacking. Validated functional assays hold the promise to accelerate the classification of these problematic variants.
